# Neurodevelopment and Risk Factors in Infants Before, During, and After the COVID-19 Pandemic in Eastern China: Cross-Sectional Study

**DOI:** 10.2196/76431

**Published:** 2025-12-15

**Authors:** Yuechong Cui, Shuting Si, Guannan Bai, Hongxing Jin, Libi Zhang, Xuying Cao, Meiying Gao, Mingyang Zou, Caihong Sun

**Affiliations:** 1 Department of Children’s and Adolescent Health Public Health College Harbin Medical University Harbin China; 2 Department of Child Health Care Yiwu Maternity and Children Hospital (Yiwu Branch of Children’s Hospital ZheJiang University School of Medicine) Yiwu China; 3 Department of Child Health Care Children's Hospital, Zhejiang University School of Medicine, National Clinical Research Center for Child Health Hangzhou China; 4 Department of Epidemiology and Health Statistics School of Child Public Health Zhejiang University Hangzhou China

**Keywords:** neurodevelopment, infants, risk factor, COVID-19, cross-sectional study

## Abstract

**Background:**

Emerging studies suggest that exposure to the COVID-19 pandemic may have heightened the risks of neurodevelopmental disorders in infants (0–1-year-old); however, population-based studies investigating these associations in Chinese contexts remain scarce, particularly including the postpandemic phase.

**Objective:**

The aim of this study was to characterize the dynamic changes in neurodevelopment among infants in eastern China during distinct phases of the COVID-19 pandemic and to identify the critical risk factors associated with infant neurodevelopmental delays.

**Methods:**

This cross-sectional study analyzes 17,621 Peabody Developmental Motor Scales-II (PDMS-II) assessments and 7877 Bayley Scales of Infant Development-Chinese Cities Revised (BSID-CR) scores of infants who visited a tertiary maternal and children hospital for routine neurodevelopment assessment from January 2019 to July 2023. Multivariate logistic regression models were used to evaluate the associations of COVID-19 pandemic phases (stage I: prepandemic, January 2, 2019, to January 22, 2020; stage II: pandemic, January 23, 2020, to December 18, 2022; and stage III: postpandemic, December 19, 2022, to July 31, 2023), seasonal variations, and perinatal variables (eg, delivery mode, birth weight, gender) with the neurodevelopmental outcomes.

**Results:**

Infants assessed at stage II of the COVID-19 pandemic had a higher risk of neurodevelopmental delay compared to infants assessed at stage I (total motor quotient: odds ratio [OR] 2.84, 95% CI 2.17-3.72; fine motor quotient: OR 2.71, 95% CI 1.99-3.68) and stage III (total motor quotient, OR 2.52, 95% CI 1.79-3.55; gross motor quotient: OR 1.65, 95% CI 1.21-2.25; fine motor quotient: OR 3.40, 95% CI 2.36-4.92). Infants assessed at stage III had the highest risk of mental development delay (OR 2.54, 95% CI 1.91-3.36). In addition, cesarean delivery, male gender, and low birth weight were independent risk factors of neurodevelopmental delay (*P*<.05).

**Conclusions:**

The COVID-19 pandemic exacerbated neurodevelopmental vulnerabilities in infants, persisting into the postpandemic period. Public health strategies should mitigate the long-term effects through early interventions.

## Introduction

The early childhood period represents a critical window of heightened neuroplasticity. However, globally, 8%-15% of children younger than 5 years have neurodevelopmental delays, which has been increasing annually [1,2]. In the United States, the prevalence rate of neurodevelopmental delay increased from 16.2% to 17.8% between 2009 and 2017 [3], while in rural China, around 30% of the infants experienced motor delays, and approximately half of the children aged 0-3 years exhibited cognitive, language, and socioemotional delays [4]. Early neurodevelopmental delay constitutes a major global public health challenge, with profound lifelong consequences such as increased risks of academic underperformance and cognitive impairment during adolescence. Longitudinal studies demonstrate that 38% of the affected children exhibit reduced labor productivity in adulthood, contributing to 1.2%-2.6% annual gross domestic product loss in low- and middle-income countries [5]. Alarmingly, 34.7% of children younger than 5 years in low-income regions failed to attain developmental potential due to intersecting poverty and neurodevelopmental impairments [5].

Early neurodevelopmental screening is a crucial public health intervention, providing children with timely access to support services [6]. Previous research consistently demonstrates that early intervention, particularly before the age of 3 years, can significantly improve outcomes for children with developmental disorders [7,8]. Neurodevelopment is influenced by a complex interplay of genetic, environmental, and social factors [9], although some factors, including perinatal factors, are amenable to intervention [10-12]. In addition, emerging evidence from high-income countries suggests that the COVID-19 pandemic has had a significant macrosocial impact on early childhood development [13,14]. Large-scale population-based data from low-income countries are also necessary due to the differences in relevant epidemic prevention policies among various countries. However, although one study from Guangzhou, China, in 2021 has demonstrated that the COVID-19 pandemic adversely affected infant neurodevelopment [15], more evidence is needed to fully understand the pandemic’s effects on children’s neurodevelopment [16].

Since 2018, the city of Yiwu, located in Zhejiang Province, China, has rolled out a comprehensive, publicly funded neurodevelopmental screening program targeting all children aged 0-3 years. This initiative is remarkable because it provides free screening services to around 3000 children each year. Such a large-scale and long-running program presents a distinctive opportunity for conducting real-world research with extensive data. Therefore, we conducted this study by using the screening data as mentioned above and aimed to evaluate the trends and characteristics of infant neurodevelopment after the implementation of such a screening program. Moreover, we aimed to identify the key associated factors for infants’ neurodevelopment and assess the influence of the COVID-19 pandemic. Findings from this study will contribute substantial evidence, which can be used to inform the formulation of public health policies and the design of interventions. These policies and interventions are crucial for promoting optimal child development in China.

## Methods

### Study Population

This cross-sectional study retrospectively analyzes infants who underwent routine neurodevelopmental assessments at Yiwu Maternity and Children’s Hospital (a tertiary grade A hospital) between January 2019 and July 2023. Yiwu, a metropolitan hub in central Zhejiang province with 1.86 million residents, served as the study setting, representing urban populations in the high-income eastern area of China. Data were extracted from 2 electronic health systems: (1) Neurodevelopmental Assessment System—providing evaluation scores, assessment dates, and living address and (2) Child Healthcare System—containing sociodemographic (eg, parental age, education) and perinatal variables (delivery mode, birthweight, gestational age at birth, date of birth, and infant’s sex).

### Ethical Considerations

This study was conducted in accordance with the Declaration of Helsinki [17] and was approved by the ethics committee of Yiwu Maternity and Children Hospital (approval 2024-Research-07). Because it utilizes a secondary analysis of pre-existing anonymized data, which had no privacy threats to individuals, and the unique ID used to link different data is actually deidentified, and the matching work was performed by the stall of data management department to ensure data security, the informed consent was waived. Given that the study did not include direct engagement with participants, no monetary compensation was offered.

### Measurements of the General and Clinical Characteristics

We extracted children’s demographic data (eg, infant’s sex, date of birth, residence) and neurodevelopmental assessment data from the Neurodevelopmental Assessment System and linked it to the Child Healthcare System via a unique ID to obtain general parental characteristics (eg, age, educational level, parity) and child birth characteristics (eg, gestational age at birth, birth weight, delivery mode, date of birth).

Parental age when the child was born was determined by subtracting the parents' birthdays from the child's birthday. Given the limited sample size for parity exceeding 4 times, we grouped these cases as “4 times and above.” Preterm birth was defined as the gestational age of less than 37 weeks at delivery. Birthweight was categorized into 3 groups: low birth weight (<2500 g), normal birth weight (2500-3999 g), and macrosomia (≥4000 g). Seasons were divided into spring (March, April, and May), summer (June, July, and August), autumn (September, October, and November), and winter (December, January, and February). Based on the living address, the residence of the participants was categorized into urban and town/rural areas. We categorized the timeline into 3 stages of the COVID-19 pandemic in Zhejiang, China: stage I (prepandemic: January 2, 2019, to January 22, 2020), stage II (pandemic: January 23, 2020, to December 18, 2022), and stage III (postpandemic: December 19, 2022, to July 31, 2023).

### Assessment of Neurodevelopment

In 2018, Yiwu was one the first municipalities in Zhejiang Province to implement a regional early childhood development screening program for infants aged 0-3 years, incorporating standardized developmental assessments into routine pediatric care at the Yiwu Maternity and Children’s Hospital. Neurodevelopmental evaluations were routinely administered to infants starting from January 2019. Therefore, we included the data starting from January 2019. Two validated instruments were administered: Peabody Developmental Motor Scales-II (PDMS-II) and Bayley Scales of Infant Development-Chinese Cities Revised (BSID-CR). PDMS-II was employed to assess the gross motor quotient (GMQ) and fine motor quotient (FMQ) for infants aged 2-6 months. The total motor quotient (TMQ) is calculated as (GMQ + FMQ)/2. BSID-CR was applied to assess the development in infants aged 8-12 months, and it encompasses 2 subscales: mental development index (MDI) and psychomotor development index (PDI) to measure cognitive/language abilities and gross/fine motor skills, respectively. There were no data of repeated measurements for the same kind of assessment. All assessments followed standardized protocols: (1) being conducted in distraction-free rooms after 10-minute infant acclimation, (2) performed by pediatric professionals certified in developmental assessment tools (interrater reliability κ>0.85), (3) double-checked data entry to minimize recording errors, and (4) corrected age was used for preterm infants.

Developmental outcomes were classified using validated cutoff criteria aligned with Chinese norms for early childhood assessment, with scores below 90 on any indicator defined as neurodevelopmental delay [18]. For the PDMS-II, scores of TMQ<90 were classified as total motor delay, encompassing both gross motor delay (GMQ<90) and fine motor delay (FMQ<90). For the BSID-CR, scores of MDI<90 indicated below-average cognitive and language development, defined as mental development delay, and scores of PDI<90 signified motor skill deficits, particularly in coordination and object manipulation, defined as psychomotor development delay. Composite neurodevelopmental delay was defined as having either MDI or PDI below 90.

### Statistical Analysis

First, we conducted a descriptive analysis. Continuous variables were assessed for normality by using Shapiro-Wilk tests. Normally distributed data were expressed as mean (SD), while nonnormally distributed data were summarized as median (IQR). Categorical variables were described using numbers and percentages. Second, we assessed the differences in the general characteristics between groups with and without neurodevelopmental delay. Two-sample independent *t* tests were applied for normally distributed data, while Mann-Whitney *U* tests were performed for nonnormally distributed data. Chi-square tests were used for categorical variables or Fisher exact tests when expected cell counts were <5. For multivariable analyses, variables showing significance indicated by *P*<.01 in univariate analyses were entered into logistic regression models. Lastly, we conducted multivariable logistic regression models to examine the potential risk factors associated with neurodevelopmental delay (defined as scores <90 on BSID-CR [MDI/PDI] or PDMS-II [TMQ]). All statistical analyses were performed using R software (version 4.2.3; The R Foundation). The statistical significance was set at *P*<.05 (2-tailed).

## Results

### Basic Characteristics of the Study Participants and Comparisons Between Groups

In this study, we included a total of 17,621 PDMS-II assessments and 7877 BSID-CR assessments. Table 1 presents the general characteristics of the parents and children. For infants assessed with the PDMS-II, 64.1% (11,294/17,621) were born during COVID-19 pandemic stage II. Among infants assessed with the BSID-CR, 64.4% (5074/7877) were born during COVID-19 pandemic stage II. Table S1 of Multimedia Appendix 1 shows the comparison of the general and clinical characteristics between infants with and without neurodevelopmental delay assessed by PDMS-II. There were statistically significant differences observed in the maternal age, paternal age, maternal education, assessment age, delivery mode, preterm status, birth season, COVID-19 pandemic stages, and residence between infants with or without neurodevelopmental delay when assessed by PDMS-II. Similarly, significant differences were found in maternal age, paternal age, assessment age, birthweight, gestational age, delivery mode, gender, preterm status, birth season, COVID-19 pandemic stages, and residence between infants with and without neurodevelopmental delay when assessed by BSID-CR (Table S2 in Multimedia Appendix 1).

**Table 1:** General characteristics of the study population.

Variable				PDMS-II^a^ (n=17,621)		BSID-CR^b^ (n=7877)
**Parental characteristics**						
	Maternal age (y), mean (SD)		30.27 (4.48)		30.06 (4.47)	
	Paternal age (y), mean (SD)		31.92 (4.98)		31.71 (4.95)	
	**Parity, n (%)**					
		1	10,771 (61.1)		5337 (67.8)	
		2	6252 (35.5)		2303 (29.2)	
		3	552 (3.1)		220 (2.8)	
		≥4	46 (0.3)		17 (0.2)	
	**Maternal education, n (%)**					
		Junior high school and below	964 (5.5)		393 (5)	
		Senior high school	6333 (35.9)		3181 (40.4)	
		College and above	9854 (55.9)		4102 (52.1)	
		Unknown	470 (2.7)		201 (2.6)	
	**Paternal education, n (%)**					
		Junior high school	945 (5.4)		395 (5)	
		Senior high school	6920 (39.3)		3496 (44.4)	
		College and above	9249 (52.5)		3764 (47.8)	
		Unknown	507 (2.9)		222 (2.8)	
**Infants' characteristics**						
	Age at check (d), mean (SD)		109.99 (20.51)		271.04 (29.82)	
	Gestational age (wk), mean (SD)		38.58 (1.54)		38.57 (1.64)	
	**Birthweight (g), mean (SD)**		3290.42 (479.57)		3279.58 (488.58)	
		Low birth weight, n (%)	848 (4.8)		432 (5.5)	
		Normal birth weight, n (%)	15,607 (89.2)		6969 (88.9)	
		Macrosomia, n (%)	1032 (5.9)		439 (5.6)	
	**Delivery mode, n (%)**					
		Vaginal delivery	9329 (52.9)		4231 (53.7)	
		Cesarean delivery	8081 (45.9)		3541 (45)	
		Unknown	211 (1.2)		105 (1.3)	
	**Child’s sex, n (%)**					
		Girl	8038 (45.6)		3551 (45.1)	
		Boy	9583 (54.4)		4326 (54.9)	
	**Preterm birth, n (%)**					
		No	16,323 (92.7)		7226 (91.8)	
		Yes	1287 (7.3)		648 (8.2)	
	**Birth season, n (%)**					
		Spring	3928 (22.3)		1803 (22.9)	
		Summer	4136 (23.5)		2060 (26.2)	
		Autumn	4904 (27.8)		2262 (28.7)	
		Winter	4653 (26.4)		1752 (22.2)	
	**COVID-19 pandemic stages, n (%)**					
		Stage I	3894 (22.1)		1094 (13.9)	
		Stage II	11,294 (64.1)		5074 (64.4)	
		Stage III	2433 (13.8)		1709 (21.7)	
	**Residence, n (%)**					
		Town	6210 (35.2)		2649 (33.6)	
		City	11,411 (64.8)		5228 (66.4)	

^a^PDMS-II: Peabody Developmental Motor Scales-II.

^b^BSID-CR: Bayley Scales of Infant Development-Chinese Cities Revised.

### Infant Neurodevelopment Trend From 2019 to 2023

**Figure 1:**
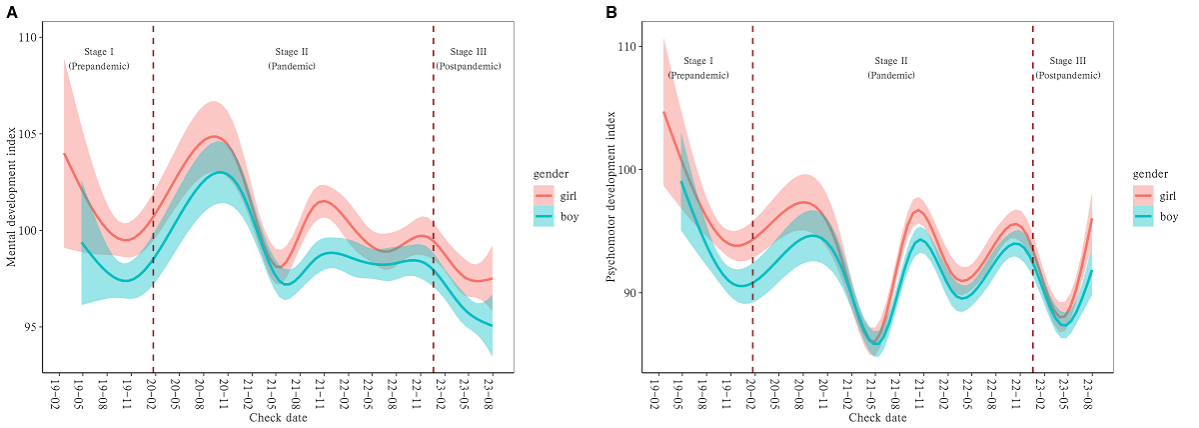
Trends in infant neurodevelopment levels in Yiwu city assessed using the Peabody Developmental Motor Scales-II from January 2019 to July 2023: (A) mental development index and (B) psychomotor index.

**Figure 2:**
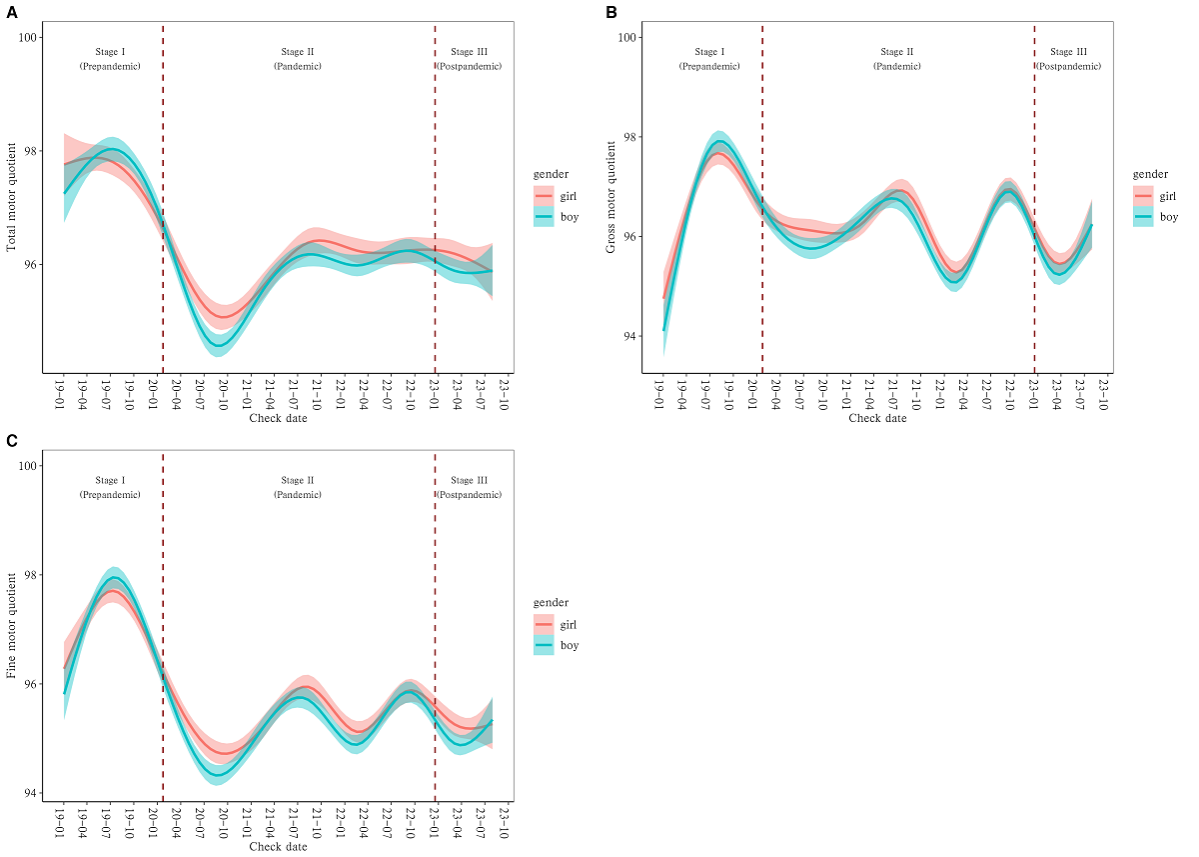
Trends in infant neurodevelopment levels, that is, (A) total motor quotient, (B) gross motor quotient, and (C) fine motor quotient in Yiwu city, assessed using the Bayley Scales of Infant Development-Chinese Cities Revised from January 2019 to July 2023.

As illustrated in Figure 1 and Figure 2, the neurodevelopmental levels of infants in Yiwu, assessed by BSID-CR and PDMS-II, exhibited fluctuations from January 2019 to July 2023. Specifically, TMQ, GMQ, and FMQ, evaluated by PDMS-II, demonstrated a downward trend from October 2019 to October 2020. Figure 1 shows that since January 2021, there has been a cyclical fluctuation with an approximate 1-year cycle, wherein TMQ showed lesser variability. Figure 2 shows that both boys and girls followed similar patterns. However, in terms of the mental and psychomotor development indices, as measured by BSID-CR, boys performed worse than girls. 

**Figure 3:**
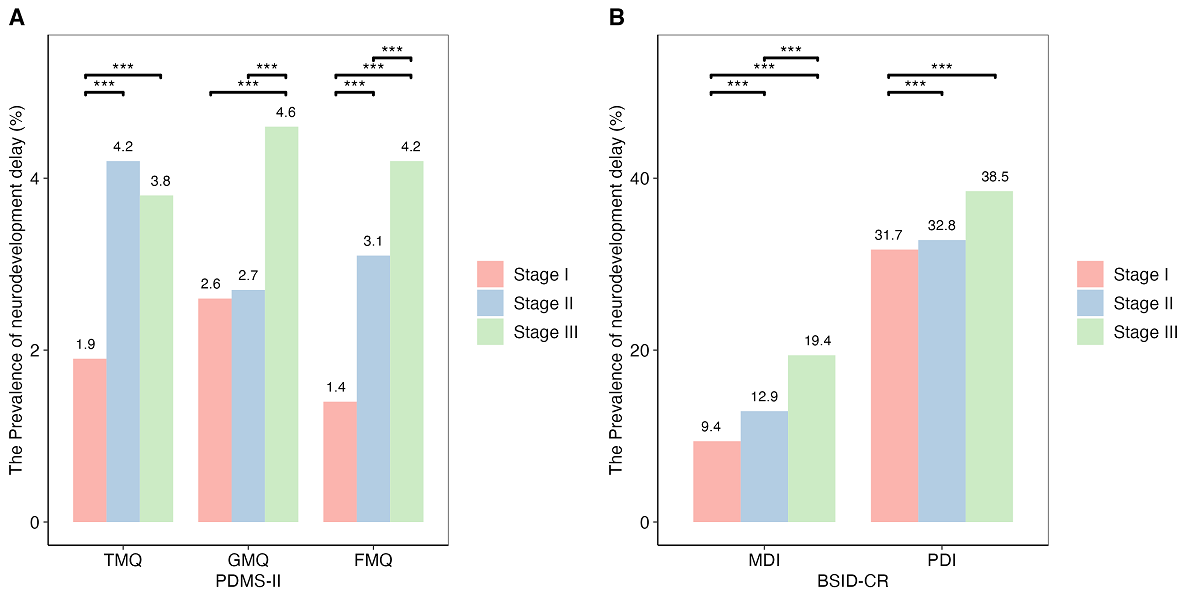
Prevalence of neurodevelopment delay in infants in Yiwu detected by (A) PDMS-II and (B) BSID-CR at different stages of the COVID-19 pandemic. BSID-CR: Bayley Scales of Infant Development-Chinese Cities Revised; FMQ: fine motor quotient; GMQ: gross motor quotient; MDI: mental development index; PDI: psychomotor development index; PDMS-II: Peabody Developmental Motor Scales-II; TMQ: total motor quotient. ***P<.05.

Figure 3 shows the prevalence of neurodevelopment delay at different stages of the COVID-19 pandemic. Across all evaluation indices (TMQ, GMQ, FMQ, MDI, PDI), stage I showed the lowest prevalence of neurodevelopmental delay.

### Factors Associated With Infant Neurodevelopment Delay

Table 2 and Table 3 present the results of logistic regression analyses in terms of the associated factors of infant neurodevelopmental delay assessed by PDMS-II and BSID-CR, respectively. Infants assessed at stage II had a higher risk of neurodevelopmental delay compared to infants assessed at stage I, even after adjusting for other variables (TMQ: odds ratio [OR] 2.84, 95% CI 2.17-3.72; FMQ: OR 2.71, 95% CI 1.99-3.68) and stage III (TMQ: OR 2.52, 95% CI 1.79-3.55; GMQ: OR 1.65, 95% CI 1.21-2.25; FMQ: OR 3.40, 95% CI 2.36-4.92). Infants assessed at stage III had the highest risk of mental development delay (OR 2.54, 95% CI 1.91-3.36). In addition, the results indicated that cesarean delivery, infant’s sex as male, preterm birth, LBW, and residing in town/rural areas were significantly associated with neurodevelopmental delay (*P*<.05). 

**Table 2:** Association between the influence factors and neurodevelopmental delay assessed by PDMS-II^a^.

Variable			TMQ^b^<90					GMQ^c^<90					FMQ^d^<90		
			OR^e^ (95% CI)		*P* value		OR (95% CI)			*P* value		OR (95% CI)			*P* value
**COVID-19 pandemic stages**															
	Stage I	Reference		Reference		Reference			Reference		Reference			Reference	
	Stage II	2.84 (2.17-3.72)		<.001		1.11 (0.86-1.43)			.43		2.71 (1.99-3.68)			<.001	
	Stage III	2.52 (1.79-3.55)		<.001		1.65 (1.21-2.25)			.001		3.40 (2.36-4.92)			<.001	
Maternal age (y)			1.00 (0.97-1.03)		.95		1.01 (0.97-1.04)			.78		1.03 (0.99-1.06)			.18
Paternal age (y)			1.01 (0.98-1.04)		.38		1.00 (0.97-1.03)			.95		0.99 (0.96-1.02)			.58
**Parity**															
	1	Reference		Reference		Reference			Reference		Reference			Reference	
	2	1.13 (0.93-1.37)		.21		0.95 (0.76-1.18)			.64		1.07 (0.86-1.32)			.55	
	3	1.51 (0.99-2.30)		.06		1.22 (0.75-1.99)			.43		1.39 (0.87-2.23)			.17	
	≥4	1.38 (0.32-5.90)		.66		—^f^			—		0.75 (0.10-5.68)			.78	
**Maternal education**															
	Junior high school	Reference		Reference		Reference			Reference		Reference			Reference	
	Senior high school	0.82 (0.58-1.15)		.25		0.69 (0.47-1.01)			.06		0.81 (0.55-1.20)			.29	
	College and above	0.77 (0.55-1.08)		.13		0.73 (0.50-1.05)			.09		0.83 (0.57-1.21)			.33	
	Unknown	0.94 (0.46-1.90)		.86		1.12 (0.56-2.25)			.74		1.29 (0.63-2.63)			.49	
Age at check (d)			1.03 (1.03-1.03)		<.001		1.01 (1.01-1.02)			<.001		1.03 (1.03-1.04)			<.001
**Delivery mode**															
	Vaginal delivery	Reference		Reference		Reference			Reference		Reference			Reference	
	Cesarean delivery	1.33 (1.12-1.58)		.001		1.33 (1.10-1.61)			.003		1.42 (1.17-1.73)			<.001	
	Unknown	1.57 (0.67-3.70)		.30		1.10 (0.40-3.05)			.85		2.04 (0.86-4.86)			.11	
**Gender**															
	Girl	Reference		Reference		Reference			Reference		Reference			Reference	
	Boy	1.11 (0.94-1.32)		.22		1.24 (1.02-1.49)			.03		1.23 (1.02-1.49)			.03	
**Preterm**															
	No	Reference		Reference		Reference			Reference		Reference			Reference	
	Yes	0.10 (0.05-0.22)		<.001		0.34 (0.20-0.56)			<.001		0.14 (0.07-0.27)			<.001	
**Birthweight**															
	Low birth weight	1.64 (0.96-2.80)		.07		2.79 (1.80-4.31)			<.001		2.44 (1.45-4.11)			<.001	
	Normal birth weight	Reference		Reference		Reference			Reference		Reference			Reference	
	Macrosomia	0.54 (0.35-0.83)		.004		0.62 (0.39-0.99)			.04		0.50 (0.30-0.82)			.006	
**Birth season**															
	Spring	Reference		Reference		Reference			Reference		Reference			Reference	
	Summer	0.87 (0.69-1.10)		.24		1.24 (0.91-1.70)			.17		1.11 (0.84-1.47)			.46	
	Autumn	0.77 (0.61-0.97)		.02		2.30 (1.75-3.03)			<.001		1.15 (0.88-1.49)			.30	
	Winter	0.47 (0.36-0.61)		<.001		1.04 (0.76-1.43)			.79		0.54 (0.40-0.74)			<.001	
**Residence**															
	City	Reference		Reference		Reference			Reference		Reference			Reference	
	Town	1.29 (1.09-1.53)		.003		1.09 (0.90-1.32)			.37		1.47 (1.21-1.78)			<.001	

^a^PDMS-II: Peabody Developmental Motor Scales-II.

^b^TMQ: total motor quotient.

^c^GMQ: gross motor quotient.

^d^FMQ: fine motor quotient.

^e^OR: odds ratio.

^f^Not available.

**Table 3:** Association between the influence factors and neurodevelopmental delay assessed by BSID-CR^a^.

Variable			Total score<90				MDI^b^<90				PDI^c^<90		
			OR^d^ (95% CI)		*P* value		OR (95% CI)		*P* value		OR (95% CI)		*P* value
**COVID-19 pandemic stages**													
	Stage I	Reference		Reference		Reference		Reference		Reference		Reference	
	Stage II	0.99 (0.84-1.17)		.93		1.45 (1.13-1.87)		.004		0.90 (0.76-1.06)		.20	
	Stage III	1.15 (0.95-1.39)		.16		2.54 (1.91-3.36)		<.001		1.00 (0.83-1.21)		.99	
Maternal age (y)			0.99 (0.97-1.01)		.35		0.99 (0.97-1.02)		.67		0.99 (0.97-1.01)		.56
Paternal age (y)			1.01 (0.99-1.03)		.18		1.02 (0.99-1.04)		.15		1.01 (0.99-1.03)		.28
Age at check (d)			1.03 (1.03-1.03)		<.001		1.03 (1.03-1.03)		<.001		1.03 (1.02-1.03)		<.001
**Delivery mode**													
	Vaginal delivery	Reference		Reference		Reference		Reference		Reference		Reference	
	Cesarean delivery	1.20 (1.07-1.34)		.001		1.12 (0.97-1.30)		.13		1.23 (1.10-1.37)		<.001	
	Unknown	1.48 (0.88-2.49)		.14		1.02 (0.48-2.21)		.95		1.72 (1.03-2.88)		.04	
**Child’s sex**													
	Girl	Reference		Reference		Reference		Reference		Reference		Reference	
	Boy	1.34 (1.20-1.49)		<.001		1.56 (1.35-1.81)		<.001		1.32 (1.18-1.47)		<.001	
**Preterm birth**													
	No	Reference		Reference		Reference		Reference		Reference		Reference	
	Yes	0.48 (0.37-0.61)		<.001		0.44 (0.31-0.64)		<.001		0.51 (0.39-0.65)		<.001	
**Birthweight**													
	Low birth weight	1.36 (1.02-1.82)		.04		1.37 (0.92-2.04)		.12		1.39 (1.04-1.86)		.03	
	Normal birth weight	Reference		Reference		Reference		Reference		Reference		Reference	
	Macrosomia	0.94 (0.75-1.19)		.61		0.79 (0.57-1.10)		.16		0.98 (0.77-1.23)		.84	
**Birth season**													
	Spring	Reference		Reference		Reference		Reference		Reference		Reference	
	Summer	1.41 (1.22-1.64)		<.001		1.36 (1.11-1.67)		.003		1.43 (1.23-1.66)		<.001	
	Autumn	0.82 (0.71-0.95)		.007		1.31 (1.06-1.62)		.01		0.72 (0.62-0.84)		<.001	
	Winter	0.48 (0.41-0.57)		<.001		1.05 (0.83-1.33)		.69		0.41 (0.35-0.49)		<.001	
**Residence**													
	City	Reference		Reference		Reference		Reference		Reference		Reference	
	Town	1.05 (0.94-1.17)		.42		1.00 (0.86-1.16)		.98		1.02 (0.91-1.14)		.69	

^a^BSID-CR: Bayley Scales of Infant Development-Chinese Cities Revised.

^b^MDI: mental development index is less than 90.

^c^Psychomotor development index is less than 90.

^d^OR: odds ratio.

## Discussion

### Principal Findings

This study uses a large-scale, retrospective dataset of a publicly funded neurodevelopmental screening program among infants since 2019 in Yiwu, China, to evaluate the early childhood neurodevelopment after the implementation of this program. Our study reveals that the COVID-19 pandemic may have adverse effects on infant neurodevelopment, persisting into the postpandemic period. The scores obtained via BSID-CR and PDMS-II assessments among infants consistently demonstrated seasonal variations. Furthermore, infant’s age, sex, birthweight, preterm birth, and mode of delivery were significantly associated with the infants' neurodevelopment. To the best of our knowledge, this study bridges a crucial evidence gap on factors influencing infant neurodevelopment by providing population-level, neurodevelopmental data spanning prepandemic, pandemic, and postpandemic periods.

This study shows that the levels of infant neurodevelopment fluctuated, especially in 2020, which might be related to the COVID-19 pandemic. Our study reveals that compared to infants in the stage I of the COVID-19 pandemic, those in stage II demonstrated significantly higher risks of motor and mental delay. One study from Guangzhou, China, in 2021 also demonstrated that the COVID-19 pandemic adversely affected infant neurodevelopment [15]. Another study conducted in New York also indicated that infants exposed and unexposed to the SARS-CoV-2 during the COVID-19 pandemic had considerably lower scores in gross motor, fine motor, and personal-social subdomains compared to historical cohorts born prior to the pandemic [19]. This adverse effect could be attributed to various factors. First, the COVID-19 pandemic and related restrictions likely limited infants' opportunities for environmental exploration and peer engagement, which could adversely impact their neurodevelopmental outcomes [15]. Second, prolonged mask-wearing may have reduced infants' exposure to facial expression recognition and other crucial social stimuli during critical developmental periods [20]. Notably, infants assessed at stage III continued to demonstrate significantly lower neurodevelopmental level compared to those in stage I. At present, there is a lack of relevant research to confirm. In our study, these infants at stage III were born to mothers who not only experienced pandemic-related stress during pregnancy but also had relatively high chance to be infected by the SARS-CoV-2. Related stress factors during pregnancy should be recognized as a potential contributing mechanism to these developmental delays [21,22]. Moreover, the infection itself might have played a role. A meta-analysis published in 2022, encompassing 8 studies with 21,419 infants, demonstrated that maternal SARS-CoV-2 infection was associated with an increased risk of fine motor impairment in offspring (OR 3.46, 95% CI 1.43-8.38) [23].

Even after adjusting for the effects of different stages of the COVID-19 pandemic on neurodevelopment, we still found several factors related to neurodevelopmental delay. In our study, we observed that boys exhibited poorer performance compared to girls not only on BSID-CR but also on PDMS-II. A cohort study conducted in Japan supported this finding, revealing a higher risk of developmental delay among boys younger than 2 years (OR 2.5, 95% CI 1.5-4.2) than girls [24]. Additionally, a 2024 review highlighted that boys are more prone to malnutrition and stunting—factors that could potentially impact neurodevelopment [25]. We hypothesized that these disparities may be linked to sex-based differences in utero exposure sensitivity. Guma and Chakravarty [26] also reported that exposure to inflammation in utero or early life can influence fetal neurodevelopment, with boys being more vulnerable than girls due to sex hormone differences. Furthermore, early language development exhibits sexual dimorphism due to genetic and hormonal factors, with girls typically developing faster than boys, leading to earlier social awareness and superior neurodevelopment levels in girls [27-29].

Our study indicates that infants with LBW exhibited poorer neurodevelopmental outcomes. This observation aligned with that reported by Wu et al [30] demonstrating infants with marginally LBW (ranging from 2000 to 2499 g) with a higher risk of neurodevelopmental delays. This may result from impaired brain connectivity and neuronal migration in LBW infants [31], where high-quality family environmental stimulation can partially mitigate the adverse neurodevelopmental impacts [32]. Consequently, timely parent-child interaction interventions should be prioritized for LBW infants to improve neurodevelopmental outcomes. Interestingly, our study shows that preterm birth appeared to be a protective factor for infant neurodevelopment. This might be a chance finding due to the limited sample size of infants with preterm birth, especially very preterm birth, in our study. Additionally, it was possible that the increased attention and care received by premature infants could have contributed to improved neurodevelopment by altering the methylation levels [33]. We recommend that future studies replicate our protocol to support or refute our findings.

We also identified cesarean delivery as a risk factor for infant neurodevelopmental delay, consistent with prior epidemiological evidence [34]. The potential harm associated with cesarean delivery may be linked to microbiome disturbances. A blinded randomized controlled trial revealed that cesarean-born infants who received vaginal microbiota transfer showed significantly better neurodevelopment at 6 months compared to those who received saline [35]. Therefore, we hypothesize that cesarean delivery may impact infant neurodevelopment by altering the microbiome disturbances.

Additionally, our study reveals seasonal variations in infants' neurodevelopment levels. We found that infants born in summer had a higher risk of mental and psychomotor development delay. This might be because most summer-born infants undergo the BSID-CR assessment in winter, when they receive less sunlight exposure. Similar to what we found, a comprehensive multi-center study encompassing Denmark, Finland, Norway, Sweden, and Western Australia has uncovered a seasonal trend in the occurrence of autism spectrum disorder [36]. This trend might be attributed to varying sunlight levels during early childhood [35]. Furthermore, recent research indicates a positive correlation between children's neurodevelopment and 25(OH)D levels [37,38]. This suggests that vitamin D, closely linked to sunlight exposure, could be a crucial factor for explaining the seasonal disparity in the neurodevelopment of infants. Further research and exploration of the underlying mechanism relationships are still needed in the future.

### Limitations

There are some limitations worthy of mention. First, as a cross-sectional design, this study could not establish causational relationships. We recommend future longitudinal follow-ups or birth cohort to replicate this study protocol in order to confirm or to refuse our findings. Second, our data, derived from a single city, may not comprehensively reflect the situation in China. It is worth noting that Yiwu serves as a pilot city for various projects, including the National Rural Early Childhood Development Project and the National Rural Revitalization Strategy-Grassroots Early Childhood Development Project, thereby possessing a certain degree of theoretical representativeness of areas with advanced child health care in China.

### Conclusions

The COVID-19 pandemic exacerbated neurodevelopmental vulnerabilities in infants, with effects persisting into the postpandemic period. Public health strategies should focus on comprehensive developmental monitoring and timely interventions for all high-risk infants to mitigate the long-term impacts.

## Appendix

Multimedia Appendix 1 Comparison of the basic characteristics of the study population with and without total motor developmental delay, assessed using Peabody Developmental Motor Scales-II.

Multimedia Appendix 2 STROBE checklist.

## Abbreviations

BSID-CR: Bayley Scales of Infant Development-Chinese Cities Revised
FMQ: fine motor quotient
GMQ: gross motor quotient
LBW: low birth weight
MDI: mental development index
OR: odds ratio
PDI: psychomotor development index
PDMS-II: Peabody Developmental Motor Scales-II
STROBE: Strengthening the Reporting of Observational studies in Epidemiology
TMQ: total motor quotient

## Data Availability

The data presented in this study are available on request from the corresponding author. The data are not publicly available because they contain information that could compromise the privacy of research participants.
