# Supplementary material for: Neurodevelopment and Risk Factors in Infants Before, During, and After the COVID-19 Pandemic in Eastern China: Cross-Sectional Study
Source: JMIR Public Health Surveill. 2025 Dec 15;11:e76431. doi: 10.2196/76431 (PMC12750074; doi:10.2196/76431)
Supplement: Multimedia Appendix 1 [file publichealth_v11i1e76431_app1.docx]

| Table S1. Comparison of the basic characteristics^a^ between total motor developmental delay assessed PDMS-II^b^. | | | |
| --- | --- | --- | --- |
| Variable | Total motor developmental delay | | *P* |
|  | No（N=16974) | Yes (N=647) |  |
| Parental characteristics |  | | |
| Maternal age, year | 30.25 ± 4.47 | 30.82 ± 4.48 | 0.002 |
| Paternal age, year | 31.89 ± 4.98 | 32.61 ± 4.95 | 0.001 |
| Parity |  |  | 0.035 |
| 1 | 10401 (61.3) | 370 (57.2) |  |
| 2 | 6008 (35.4) | 244 (37.7) |  |
| 3 | 521 (3.1) | 31 (4.8) |  |
| ≥4 | 44 (0.3) | 2 (0.3) |  |
| Maternal education |  |  | 0.023 |
| Junior high school | 914 (5.4) | 50 (7.7) |  |
| Senior high school | 6086 (35.9) | 247 (38.2) |  |
| College and above | 9520 (56.1) | 334 (51.6) |  |
| unknown | 454 (2.7) | 16 (2.5) |  |
| Paternal education |  |  | 0.129 |
| Junior high school and below | 902 (5.3) | 43 (6.6) |  |
| Senior high school | 6647 (39.2) | 273 (42.2) |  |
| College and above | 8935 (52.6) | 314 (48.5) |  |
| unknown | 490 (2.9) | 17 (2.6) |  |
| Infants' characteristics |  | | |
| Check age, day | 109.39 ± 19.78 | 125.95 ± 30.55 | <0.001 |
| Gestational age, week | 38.58 ± 1.55 | 38.54 ± 1.31 | 0.516 |
| Birthweight, gram | 3290.97 ± 481.24 | 3276.03 ± 433.38 | 0.439 |
| Birthweight |  |  | 0.013 |
| Low birth weight | 828 (4.9) | 20 (3.1) |  |
| Normal birth weight | 15014 (89.1) | 593 (92.8) |  |
| Macrosomia | 1006 (6.0) | 26 (4.1) |  |
| Delivery mode |  |  | 0.017 |
| Vaginal delivery | 9020 (53.1) | 309 (47.8) |  |
| Cesarean section | 7749 (45.7) | 332 (51.3) |  |
| unknown | 205 (1.2) | 6 (0.9) |  |
| Gender |  |  |  |
| Girl | 7763 (45.7) | 275 (42.5) | 0.114 |
| Boy | 9211 (54.3) | 372 (57.5) |  |
| Preterm |  |  | <0.001 |
| No | 15686 (92.5) | 637 (98.5) |  |
| Yes | 1277 (7.5) | 10 (1.5) |  |
| Birth season |  |  | <0.001 |
| Spring | 3746 (22.1) | 182 (28.1) |  |
| Summer | 3981 (23.5) | 155 (24.0) |  |
| Autumn | 4700 (27.7) | 204 (31.5) |  |
| Winter | 4547 (26.8) | 106 (16.4) |  |
| COVID-19 pandemic stages |  |  | <0.001 |
| Stage I | 3819 (22.5) | 75 (11.6) |  |
| Stage II | 10815 (63.7) | 479 (74.0) |  |
| Stage III | 2340 (13.8) | 93 (14.4) |  |
| Residence |  |  | <0.001 |
| Twon | 5924 (34.9) | 286 (44.2) |  |
| City | 11050 (65.1) | 361 (55.8) |  |
| ^a^ Values presented in this table are means, standard deviations, numbers and percentages. | | | |
| ^b^PDMS-II, Peabody Developmental Motor Scales-II | | | |

| Supplementary Table S2. Comparison of the basic characteristics^a^ between total neurodevelopmental delay assessed by BSID-CR^b^. | | | |
| --- | --- | --- | --- |
| Variable | Total neurodevelopmental delay | | *P* |
|  | No（N=5002) | Yes (N=2875) |  |
| Parental characteristics |  |  |  |
| Maternal age, year | 29.98 ± 4.49 | 30.20 ± 4.43 | 0.031 |
| Paternal age, year | 31.60 ± 5.01 | 31.90 ± 4.85 | 0.012 |
| Parity |  |  | 0.284 |
| 1 | 3410 (68.2) | 1927 (67.0) |  |
| 2 | 1432 (28.6) | 871 (30.3) |  |
| 3 | 149 (3.0) | 71 (2.5) |  |
| ≥4 | 11 (0.2) | 6 (0.2) |  |
| Maternal education |  |  | 0.323 |
| Junior high school and below | 251 (5.0) | 142 (4.9) |  |
| Senior high school | 1984 (39.7) | 1197 (41.6) |  |
| College and above | 2633 (52.6) | 1469 (51.1) |  |
| unknown | 134 (2.7) | 67 (2.3) |  |
| Paternal education |  |  | 0.397 |
| Junior high school and below | 244 (4.9) | 151 (5.3) |  |
| Senior high school | 2192 (43.8) | 1304 (45.4) |  |
| College and above | 2426 (48.5) | 1338 (46.5) |  |
| unknown | 140 (2.8) | 82 (2.9) |  |
| Infants' characteristics |  |  |  |
| Check age, day | 262.45 ± 24.52 | 285.97 ± 32.23 | <0.001 |
| Gestational age, week | 38.60 ± 1.62 | 38.51 ± 1.67 | 0.016 |
| Birthweight, gram | 3289.45 ± 479.16 | 3262.37 ± 504.19 | 0.018 |
| Birthweight |  |  | 0.850 |
| Low birth weight | 270 (5.4) | 162 (5.7) |  |
| Normal birth weight | 4436 (89.0) | 2533 (88.6) |  |
| Macrosomia | 276 (5.5) | 163 (5.7) |  |
| Delivery mode |  |  | 0.001 |
| Vaginal delivery | 2767 (55.3) | 1464 (50.9) |  |
| Cesarean section | 2171 (43.4) | 1370 (47.7) |  |
| unknown | 64 (1.3) | 41 (1.4) |  |
| Gender |  |  | <0.001 |
| Girl | 2366 (47.3) | 1185 (41.2) |  |
| Boy | 2636 (52.7) | 1690 (58.8) |  |
| Preterm |  |  | 0.006 |
| No | 4556 (91.1) | 2670 (92.9) |  |
| Yes | 444 (8.9) | 204 (7.1) |  |
| Birth season |  |  | <0.001 |
| Spring | 1113 (22.3) | 690 (24.0) |  |
| Summer | 1084 (21.7) | 976 (33.9) |  |
| Autumn | 1510 (30.2) | 752 (26.2) |  |
| Winter | 1295 (25.9) | 457 (15.9) |  |
| COVID-19 pandemic stages |  |  | <0.001 |
| Stage I | 724 (14.5) | 370 (12.9) |  |
| Stage II | 3274 (65.5) | 1800 (62.6) |  |
| Stage III | 1004 (20.1) | 705 (24.5) |  |
| Residence |  |  | <0.001 |
| Twon | 1611 (32.2) | 1038 (36.1) |  |
| City | 3391 (67.8) | 1837 (63.9) |  |
| ^a^ Values presented in this table are means, standard deviations, numbers and percentages. | | | |
| ^b^BSID-CR: Bayley Scales of Infant Development-Chinese Cities Revised | | |  |
| Total neurodevelopmental delay, at least one of the mental development and Psychomotor development indexes is less than 90. | | | |
